# How the Experimental Setting Influences Representativeness: A Review of Gaze Behavior in Football Penalty Takers

**DOI:** 10.3389/fpsyg.2018.00682

**Published:** 2018-05-08

**Authors:** Johannes Kurz, Jörn Munzert

**Affiliations:** Neuromotor Behavior Laboratory, Department of Psychology and Sports Sciences, Justus-Liebig-University Giessen, Giessen, Germany

**Keywords:** football, penalty, far-aiming task, gaze behavior, experimental conditions

## Abstract

This article reviews research on the gaze behavior of penalty takers in football. It focuses on how artificial versus representative experimental conditions affect gaze behavior in this far-aiming task. Findings reveal that—irrespective of the representativeness of the experimental conditions—different instructions regarding the aiming strategy and different threat conditions lead to different gaze patterns. Results also reveal that the goal size and the distance to the goal did not affect the gaze behavior. Moreover, it is particularly run-up conditions that lead to differences. These can be either artificial or more natural. During a natural run-up, penalty takers direct their gaze mainly toward the ball. When there is no run-up, they do not direct their gaze toward the ball. Hence, in order to deliver generalizable results with which to interpret gaze strategies, it seems important to use a run-up with a minimum length that is comparable to that in a real-life situation.

## Introduction

Perception–action coupling is a promising field of study offering new insights into sensorimotor control. This article reviews studies focusing on gaze behavior in football penalty takers and how the representativeness of the experimental setting influences gaze behavior in this far-aiming task. The idea of creating representative task designs has been an important issue in experimental psychology for many years (Brunswick, 1956) and has been introduced to the field of perception-action coupling (e.g., Dicks et al., 2009, 2010). It has been suggested that task designs should represent the organism’s natural environment (see Araújo et al., 2007, for details) and that task designs should comprise representative stimuli and allow participants to respond with unrestricted movements. Both aspects play an essential role when studying perception and action. Dicks et al. (2009) suggested that representative experimental conditions are mandatory to gain generalizable conclusions. Several studies have supported this suggestion by showing how representativeness affects performance and gaze behavior. For example, Mann et al. (2010) have shown that cricket batsmen’s performance was better in representative experimental conditions compared to artificial experimental conditions. Another study by Dicks et al. (2010) found that football goalkeepers’ performance and gaze behavior differed between artificial and representative experimental conditions: In representative experimental conditions, performance was better and gaze was directed toward the ball earlier and for a longer duration. Studies on walking in real life using a mobile eye tracker compared to watching videos of walking also found significantly different patterns of gaze behavior (’t Hart et al., 2009; Foulsham et al., 2011): In real life, gaze was more centralized due to participants making head movements instead of large saccades, and gaze was directed more toward near objects and toward the path they were walking on. Additionally, results have revealed that the visual angle is smaller when watching videos compared to real-life conditions. This leads to restrictions of head movements. These restrictions lead, in turn, to limitations in gathering further information (e.g., vestibular and other crossmodal information). Furthermore, in cycling Zeuwts et al. (2016) found that gaze is directed more toward the path in real life than in the laboratory. Such findings reveal the need to create representative experimental conditions to investigate gaze behavior in its natural environment (see Land and McLeod, 2000; Hayhoe and Ballard, 2005). They emphasize that gaze behavior is task-specific (Yarbus, 1967), and, moreover, that it is based on a just-in-time mechanism when examined under natural interactive conditions (Ballard et al., 1995; ’t Hart et al., 2009).

The present review focuses on gaze behavior in football penalties. This far-aiming task is of paramount importance in football. The specificity of this task is that it comprises two different task-related goals: the ball (proximal goal) that has to be hit with high precision and the corner (distal goal) where the ball has to be placed successfully (Kurz et al., 2018). A third area of interest for gaze behavior is defined by the goalkeeper who tries to prevent the penalty taker from scoring a goal. There have been discussions regarding whether either observing or ignoring the goalkeeper’s reaction is the more successful strategy (van der Kamp, 2006). Although research shows that instructions for both strategies can indeed influence gaze behavior, the keeper-independent strategy has proven to be the more successful one (Noël and van der Kamp, 2012). However, up to now, no study has investigated gaze behavior in open-play situations, but only in artificial or representative conditions (McGuckian et al., 2018). Therefore, several aspects that might effect performance and gaze behavior have not been studied so far (e.g., minute of play, current score, presence of spectators, or goalkeeper characteristics). As a result, we did not consider these aspects in the present review. We review research on the gaze behavior of penalty takers focusing on how gaze behavior in this far-aiming task is affected by artificial versus representative experimental conditions. The aim of the present review is to deliver support for the need to reinterpret data on gaze behavior in artificial experimental conditions and to emphasize the need for research in visual science to be carried out under representative experimental conditions.

## Literature Search

**FIGURE 1:**
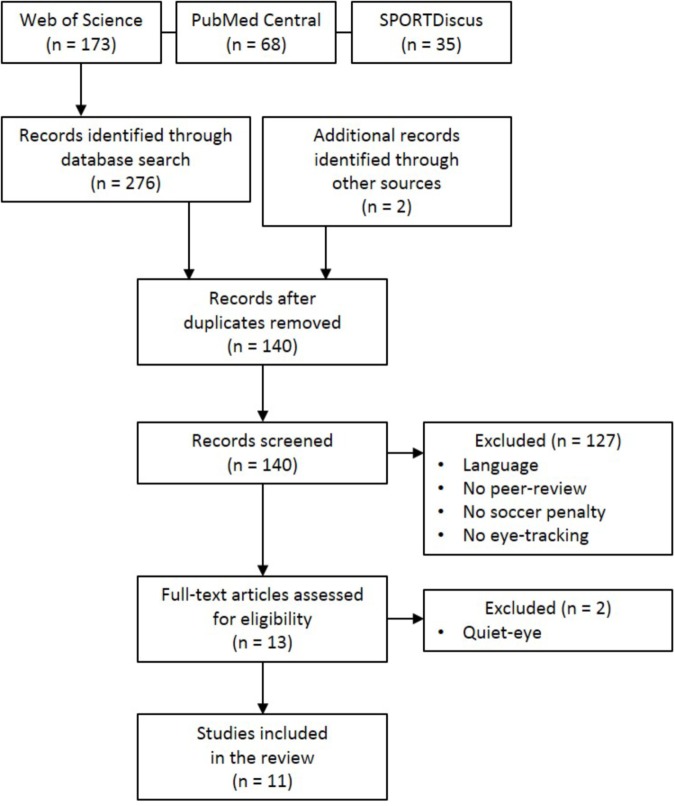
Flow diagram depicting the selection of relevant literature from identification to final inclusion of studies examine gaze behavior in football penalty takers.

We searched for literature in the following electronic databases (**Figure 1**): *Web of Science*, *PubMed Central*, and *SPORTDiscus*. Within each database, we used the keyword *penalty* combined with one of the following four keywords: *eye tracking*, *gaze behavior*, *eye movement*, or *visual search*. We also examined the references in relevant articles. Studies were included when (1) the task was to shoot a penalty in football, (2) gaze behavior was recorded, and (3) the article was written in English. We manually excluded studies addressing the so-called “quiet-eye” phenomenon (e.g., Vickers, 2007) because these focus mainly on the last fixation and do not take gaze behavior during the complete run-up into account. **Table 1** reports relevant descriptive information on the included studies.

**Table 1:** Overview of studies on gaze behavior of penalty takers in football.

Author(s) (year)	Real Goalkeeper	Run-up	Resulting categorization	Visual angle [°]	Goal size [m]	Distance [m]	Ball
Bakker et al., 2006	No	No	Artificial	36.80	1.65 × 0.55	2.48	Foam ball
Binsch et al., 2008	No	No	Artificial	38.92	2.0 × 0.81	2.83	Foam ball
Binsch et al., 2010	No	No	Artificial	38.92	2.0 × 0.81	2.83	Size 4
van der Kamp, 2011	No	2 m	Artificial	36.52	2.27 × n/a	3.44	Foam ball
Wilson et al., 2009	Yes	One step	Artificial	39.60	3.6 × 1.2	5.0	Size 5
Wood and Wilson, 2010b	Yes	One step	Artificial	39.60	3.6 × 1.2	5.0	Size 5
Timmis et al., 2014	Yes	Individual	Representative	33.92	3.66 × 1.83	6.0	Size 4
Noël and van der Kamp, 2012	Yes	Individual	Representative	31.05	5.0 × 2.0	9.0	Size 5
Wood and Wilson, 2010a	Yes	Individual	Representative	36.81	7.32 × 2.44	11.0	Size 5
Kurz et al., 2018	Yes	Individual	Representative	36.81	7.32 × 2.44	11.0	Size 5
Hüttermann et al., 2014	Yes	> 3.5 m	Representative	36.81	7.32 × 2.44	11.0	Size 5


We are well aware that a dichotomy between artificial and representative experimental conditions does not exist (e.g., Hadlow et al., 2018), but we prefer to treat this problem as a continuum with experimental conditions closer to one end of an artificial–representative dimension. To define studies using artifical and studies using representative experimental conditions, we performed a two-step categorization: In the first step, we defined conditions that include penalty shots toward a goal with a real goalkeeper who tries to prevent the penalty taker from scoring. In a second step, we defined conditions in which a minimum length of run-up should be given. The second step was defined to study the impact of the proximal and the distal goal. We categorized the situation as representative only when a run-up involved more than one step. Our argument was that only then is the ball relevant as the proximal goal. These two categories correspond with two categories defined by McGuckian et al. (2018): (1) *laboratory in situ* where participants were allowed to move freely, but non-live stimuli were presented and (2) *controlled in-situ* where participants were allowed to move freely and live stimuli were presented.

## Gaze Behavior in Football Penalty Takers

Based on this two-step categorization (**Table 1**), we defined studies as either artificial or representative. We then arranged the studies in ascending order according to goal size. As **Table 1** shows, the goal size and the distance between the goal and the penalty spot are interdependent. This is because most studies chose a visual angle between the penalty spot and both goalposts that is similar to the visual angle (∼36°) in a real-life situation (goal size: 7.32 × 2.44; distance: 11 m). Furthermore, studies using artificial experimental conditions applied a mean goal size of 2.40 × 0.85 m and a mean distance of 3.4 m (*SD* = 1.1 m). In contrast, studies using representative experimental conditions applied a mean goal size of 6.12 x 2.23 m and a mean distance of 9.6 m (*SD* = 2.2 m).

The first study (Bakker et al., 2006) examining the gaze behavior in football penalties used artificial experimental conditions. Penalty takers had to shoot toward a screen onto which a goal and a goalkeeper were projected. Penalty takers did not perform a run-up and they were asked to follow three different instructions: (1) to shoot as well as possible, (2) to shoot as well as possible and make sure to attend to the goalkeeper, and (3) to shoot as well as possible and make sure to hit the open space. When penalty takers shot according to Instruction 1, they directed their gaze for about 38% of the trials toward the goalkeeper and for about 59% toward the open space within the goal. Under Instruction 2, they directed their gaze for about 77% of the trials toward the goalkeeper and for about 22% toward the open space. In contrast, under Instruction 3, they directed their gaze for about 20% of the trials toward the goalkeeper and for about 79% toward the open space. In two further studies by Binsch et al. (2008, 2010) using artificial experimental conditions, penalty takers were asked to shoot toward a goal projected onto a screen. There was no run-up and penalty takers were asked (1) to shoot as accurately as possible, (2) to shoot as accurately as possible and not to shoot within the reach of the goalkeeper, and (3) to shoot as accurately as possible and to shoot into the open space within the goal. Results revealed that, irrespective of instructions, penalty takers first directed their gaze toward the goalkeeper and afterward toward the open space within the goal until they hit the ball. Some participants directed their gaze toward the goalkeeper again shortly before they hit the ball. Wilson et al. (2009) asked penalty takers to shoot toward a goal with a real goalkeeper using one step as run-up. Penalty takers were instructed to shoot toward the areas of the goal where they expected the best chance of scoring under a low-threat and high-threat condition. Results showed no significant differences for total number of fixations between the locations goalkeeper and open space within the goal irrespective of threat conditions. However, gaze was directed significantly longer toward the goalkeeper (*M* = 3.9 s) compared to the open space within the goal (*M* = 1.9 s) irrespective of threat conditions. In another study by Wood and Wilson (2010b) using artificial experimental conditions, penalty takers had to shoot toward a goal with a real goalkeeper. However, they were allowed to perform only one step as run-up. In this study, penalty takers were instructed to score as many goals as possible. Three typical gaze strategies were instructed: (1) to ignore the goalkeeper’s reaction, (2) to ignore the goalkeeper’s reaction and to direct their gaze toward the opposite corner from that to which they intended to aim the ball, and (3) to observe the goalkeeper’s reaction. Results showed no significant differences between conditions, though the duration of last fixation was shorter in Instruction 1 (*M* = 223 ms) compared to Instruction 2 (*M* = 317 ms) and Instruction 3 (*M* = 329 ms). No results were provided on which locations penalty takers directed their gaze toward. In a further study by van der Kamp (2011), penalty takers had to shoot toward a screen on which a goal and a goalkeeper were projected. Penalty takers were asked to score a goal and to shoot the ball toward the opposite side to that toward which the goalkeeper dived. In contrast to the aforementioned studies in which penalty takers performed no or only one step as run-up, here, penalty takers were required to take exactly 2 s and the start of the run-up was 2 m behind the ball. In the first section of the run-up, gaze was directed mainly toward the goalkeeper’s upper and lower body. However, during the last section, gaze was directed mainly toward the open space within the goal and toward the floor (including the ball).

In a study by Timmis et al. (2014) using representative experimental conditions, penalty takers had to shoot a ball toward a goal with a real goalkeeper and run up individually. Penalty takers were asked to score as many goals as possible, to avoid attempting to deceive the goalkeeper, and to take either a placement or a power penalty. Irrespective of taking a placement or a power penalty, penalty takers directed their gaze mainly toward the ball (*M* = 63%) and less toward the goalkeeper (*M* = 6%) and less toward the open space within the goal (*M* = 13%). Noël and van der Kamp (2012) asked penalty takers to shoot toward a goal with a real goalkeeper, and the run-up was a matter of individual choice. At the beginning of the task, penalty takers directed their gaze mainly toward the goalkeeper, toward the open space within the goal, and toward the ball (which seems to be necessary for a spatial calibration for the run-up; see Kurz et al., 2018). Closer to foot–ball contact, penalty takers then directed their gaze almost exclusively toward the ball. This gaze pattern appears to be independent from the penalty takers’ strategy of either ignoring or observing the goalkeeper’s reaction. When penalty takers tried to ignore the goalkeeper’s reaction, the time gaze was directed toward the ball increased from about 21% at the beginning of the task to about 90% just before foot–ball contact. When penalty takers tried to observe the goalkeeper’s reaction, the time gaze was directed toward the ball increased from about 5% at the beginning of the task to about 46% just before foot–ball contact. In another study by Wood and Wilson (2010a), penalty takers were asked to shoot a ball toward a goal with a real goalkeeper and run up individually. Penalty takers were also asked to do their best in a low- and a high-threat condition. Results showed that during the aiming phase, penalty takers distributed their gaze between the goalkeeper and the open space within the goal. Additionally, in the high-threat condition (*M* = 462 ms), gaze was directed longer toward the open space within the goal than in the low-threat condition (*M* = 347 ms). During the run-up, gaze was directed exclusively toward the ball (*M* = 430 ms) and not toward the goalkeeper (*M* = 0 ms) or the open space within the goal (*M* = 0 ms). Similar results were found by Kurz et al. (2018) when penalty takers had to shoot toward a goal with a real goalkeeper and run up individually while ignoring the goalkeeper’s reaction. At the beginning of the task, penalty takers distributed their gaze mainly between the goalkeeper, the open space within the goal, and the ball. Closer to foot–ball contact, they directed their gaze almost exclusively toward the ball. When the goalkeeper tried to save the ball, penalty takers directed their gaze toward the ball for about 45% of the time at the beginning of the task and for about 70% during the last three steps. Gaze was hardly ever directed toward the open space within the goal (*M* = 4%) during the last three steps. Using representative experimental conditions, Hüttermann et al. (2014) asked penalty takers to shoot toward the side opposite to the one the goalkeeper dived toward and to score as many goals as possible. Penalty takers had to shoot toward a goal with a real goalkeeper and they were required to start their run-up at least 3.5 m behind the ball. Penalty takers received two different instructions concerning their gaze behavior: (1) a condition in which they received no further instruction and (2) a condition in which they were instructed to direct their gaze toward a 1 × 1 m area between the ball and the goalkeeper. In compliance with Instruction 2, penalty takers directed their gaze toward the 1 × 1 m area. Under Instruction 1, penalty takers mainly distributed their gaze between the ball, the goalkeeper, and the open space within the goal on most trials (77%).

## Summary and Conclusion

In recent years, research on the gaze behavior of penalty takers in football has become an interesting topic. A series of studies has been carried out to gain a better understanding of penalty takers’ gaze behavior. The aim of this article was to review research on the gaze behavior of penalty takers in football and focus on research on how artificial versus representative experimental conditions affect gaze behavior in this far-aiming task. Furthermore, we aimed to deliver support for a reinterpretation of data on gaze behavior in artificial compared to representative experimental conditions.

The first and foremost question is whether participants performed the same task in the aforementioned studies. Most studies applied different experimental settings, such as different goal sizes, distances, balls, and lengths of run-up. Therefore, one could argue that participants performed different tasks. However, irrespective of these differences, all studies applied a similar visual angle (*M* = 36.9°, *SD* = 2.5°) ensured by adjusting the distance to the goal size. Thus, a smaller goal size resulted in a smaller distance and vice versa. Furthermore, in each study, participants were asked to shoot a ball toward a target within a goal; and in some studies, a real goalkeeper tried to prevent the participants from scoring a goal. Therefore, we argue that participants had to perform a similar task, and this justifies comparing the studies.

As outlined above, results showed differences in the gaze behavior of penalty takers depending on whether studies used artificial or representative experimental conditions. In studies with artificial experimental conditions, penalty takers directed their gaze mainly toward the goalkeeper and the open space within the goal. In studies with representative experimental conditions, penalty takers distributed their gaze between the goalkeeper, the open space within the goal, and the ball during the preparation phase. During the last three steps gaze was directed mainly toward ball. Gaze was even directed toward the ball when penalty takers were instructed explicitly to observe the goalkeeper’s reaction (Noël and van der Kamp, 2012). Thus, we suggest that this gaze pattern shown in studies using representative experimental conditions can be considered to be generalizable. Furthermore, we suggest that findings from studies using artificial experimental conditions cannot be compared to the preparation or the execution phase from studies using representative experimental conditions. It can be argued that the differences in gaze behavior depend on whether or not penalty takers perform a run-up irrespective of the presence of a real goalkeeper (van der Kamp, 2011; Noël and van der Kamp, 2012). When penalty takers did not perform a run-up, their position in relation to the ball was constant and, as a consequence, they did not have to refresh the relative position of the ball. This is one possible explanation why they did not direct their gaze toward the ball. In contrast, when penalty takers performed a run-up, their relative position to the ball changed, and they therefore had to refresh the relative position of the ball continuously. This seems to be necessary in order to obtain optimal foot–ball contact (Kurz et al., 2018). We suggest that this is the reason why gaze behavior changed during the run-up and why penalty takers directed their gaze almost exclusively toward the ball the closer they came to foot–ball contact.

Dicks et al. (2010) and Mann et al. (2010) have already shown that representative experimental conditions are mandatory to gain generalizable conclusions on performance environments. They demonstrated that findings from artificial and highly controlled experimental conditions are unlikely to be comparable with findings from more natural and less controlled experimental conditions. Thus, we suggest that future studies should consider this point and try to create representative experimental conditions (Dicks et al., 2009). These should include a run-up of a minimum length. Furthermore, we suggest that results on gaze behavior gained from studies using artificial experimental conditions without a run-up should be interpreted with caution, because these studies overestimate the number and the duration of fixations focused on the goalkeeper and the open space within the goal. Recently, Cañal-Bruland and Mann (2015) extended this approach by arguing that future studies should also consider situational and contextual (non-kinematic) information. However, it seems to be a real challenge to consider these important aspects in controlled experimental conditions.

In addition to differences in gaze behavior, we also identified similarities between studies using artificial and studies using representative experimental conditions. As shown repeatedly, different instructions on the same task result in different gaze patterns (Yarbus, 1967). This has also been shown for the gaze behavior of penalty takers in football irrespective of experimental conditions. For example, Bakker et al. (2006) used artificial experimental experimental conditions and Noël and van der Kamp (2012) used representative experimental conditions to show that penalty takers’ gaze was directed more toward the goalkeeper when they were asked to observe the goalkeeper’s reaction. In contrast, when penalty takers were asked to ignore the goalkeeper’s reaction, they directed their gaze less toward the goalkeeper. However, all other studies applied a huge number of different instructions to manipulate the gaze behavior. These studies reveal that gaze behavior of football penalty takers can be influenced by instructions. In particular, findings from studies using representative experimental conditions showed that gaze behavior of football penalty takers is task-specific and that it is based on a just-in-time mechanism. Another similarity is that irrespective of the experimental conditions, penalty takers made more fixations and directed their gaze longer toward task-relevant locations in high-threat conditions compared to low-threat conditions (Wilson et al., 2009; Wood and Wilson, 2010a). This has been found irrespective of whether or not a real goalkeeper was present and whether or not penalty takers performed a run-up.

Furthermore, findings from studies using representative experimental conditions showed that differences in goal size and distance do not affect gaze behavior. For example, Timmis et al. (2014) applied a goal size of 3.66 x 1.83 m and a distance of 6 m; Noël and van der Kamp (2012), a goal size of 5.0 x 2.0 m and a distance of 9 m; and Kurz et al. (2018), a goal size of 7.32 x 2.44 m and a distance of 11 m resulting in a mean visual angle of 33.9° (33.9°, 31.0°, and 36.8°, respectively). Additionally, these studies also used different instructions. However, results revealed that prior to the beginning of the run-up, gaze was distributed between the goalkeeper, the goal, and the ball; and during the run-up, gaze was directed mainly toward the ball. Based on these findings it remains unclear whether differences in goal size and distance affect shooting performance.

Finally, some other aspects were not considered due to the limited number of studies. For example, we did not review whether the expertise level of the participants resulted in different gaze behavior because 10 out of 11 studies had recruited university or intermediate football players with a mean experience of playing football on a competitive level for 12.9 years (*SD* = 2.2). Only one study (van der Kamp, 2011) compared different expertise levels in the participants. Furthermore, we did not review other aspects such as anxiety, environmental conditions, and knowledge of the opponent, because such aspects have not been studied so far.

In general, this review provides further insight into how the artificial versus representative distinction—and particularly whether participants had to perform a run-up—impacts on the interpretation of gaze strategies in studies on football penalties. We identified the length of the run-up as key feature which influences gaze behavior even if we have to consider that the length of the run-up is correlated with other features such as goal size or distance. In general, the review shows that gaze behavior in studies using artificial or representative experimental conditions differs. Even if the basic task, i.e., shooting a ball toward a target within the goal, seems to be the same, we would still argue that the task is modified substantially when reducing the run-up to a minimum. The essential task characteristic that changes in most artificial conditions is the reduced difficulty to obtain an optimal foot–ball contact. Thus, we suggest that results from studies using artificial experimental conditions are hardly comparable with studies using representative experimental conditions. Furthermore, we suggest that future studies should apply a minimum length of a run-up (more than one step).

## Author Contributions

JK made substantial contributions to the literature search, conception, writing, and interpretation of data. JM made substantial contributions to the conception, writing, and interpretation of data. JK and JM participated in drafting the article and revising it critically for important intellectual content; and gave final approval of the version to be submitted and agreed to be accountable for all aspects of the work in ensuring that questions related to the accuracy or integrity of any part of the work are appropriately investigated and resolved.

## Conflict of Interest Statement

The authors declare that the research was conducted in the absence of any commercial or financial relationships that could be construed as a potential conflict of interest.
